# Unusual Presentation of Cutaneous Leishmaniasis: Ocular Leishmaniasis

**DOI:** 10.1155/2017/3198547

**Published:** 2017-01-22

**Authors:** Masoud Doroodgar, Moein Doroodgar, Abbas Doroodgar

**Affiliations:** ^1^School of Medicine, Shahid Beheshti University of Medical Sciences, Tehran, Iran; ^2^Department of Medical Parasitology, Kashan University of Medical Sciences, Kashan, Iran

## Abstract

The leishmaniases are parasitic diseases that are transmitted to humans by infected female sandflies. Cutaneous leishmaniasis (CL) is one of 3 main forms of the disease. CL is the most common form of the disease and is endemic in many urban and rural parts of Iran and usually caused by two species of* Leishmania*:* L. major* and* L. tropica.* We report a case of unusual leishmaniasis with 25 lesions on exposed parts of the body and right eyelid involvement (ocular leishmaniasis). The patient was a 75-year-old male farmer referred to health care center in Aran va Bidgol city. The disease was diagnosed by direct smear, culture, and PCR from the lesions. PCR was positive for* Leishmania major.*

## 1. Introduction

Leishmaniasis is a disease caused by protozoan parasites of the genus* Leishmania* and transmitted by the bite of infected female phlebotomine sandflies. The disease can be seen in three forms: cutaneous, mucocutaneous, and visceral leishmaniasis [1–3]. Leishmaniasis is endemic in 98 countries with more than 350 million people at risk. Leishmaniasis is associated with environmental changes such as deforestation, building of dams, irrigation schemes, and urbanization [4]. The cutaneous leishmaniasis (CL) with 1.5 million new cases per year is the most common form of the disease and causes skin lesions, mainly ulcers, on exposed parts of the body [3, 4].

In the old and new world, up to 90% of cases of CL occur in Afghanistan, Algeria, Iran, Saudi Arabia, and Syrian Arab Republic and in Bolivia, Brazil, Colombia, Nicaragua, and Peru [3]. Both form of rural and urban CL; zoonotic CL (ZCL) and anthroponotic CL (ACL) were seen in Iran. CL with about annually 20,000 new cases is considered one of the most important parasitic diseases in Iran. In addition, sometimes there are a few unusual and atypical clinical forms [5–10].

Here a case of unusual leishmaniasis (ocular leishmaniasis) from endemic area of Aran va Bidgol (Isfahan province, central Iran) with 25 lesions on exposed parts of the body and eight lesions on the face and right eyelid involvement (OL) is reported. The disease was diagnosed by direct smear, culture of lesion, and RAPD-PCR technique for identifying the leishman agent causative. The PCR product was compared to reference stocks:* L. major* (MHOM/IR/75/ER) and* L. tropica* (MHOM/IR/IR/99) and the results were obtained. The data related to the patient was analyzed by descriptive statistics and bands of PCR product were compared to the standard marker (XIV) strains.

## 2. Case Report

A 75-year-old man with complaint of lesions extended on the hands, legs, face, and upper lid of right eye was referred to a health center of Kashan.

He was a farmer in Aran va Bidgol city from Isfahan province in the center of Iran. The patient about his infection said, sandflies have bitten him when referring to the farm for irrigation on the nights in the season of sandflies activity.

**Figure 1:**
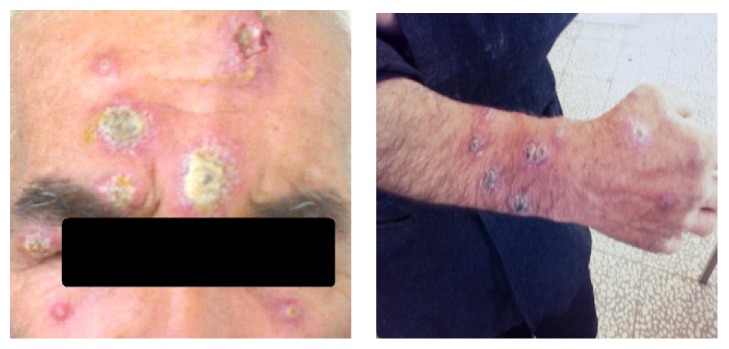
Patient with eight lesions on the face, right eyelid, and some lesions on the hands.

Based on questioning, the patient described the appearance of 25 multiple nodular, ulcerative, and crusted lesions on his body. In physical examination the size of lesions was varied from a few millimeters to several centimeters. He had no particular medical history and systemic symptoms. There were eight lesions on the face that one of them was on the upper eyelid right eye (Figure 1).

In fact, the patient suffered from ocular leishmaniasis in addition to having multiple CL lesions. He had no fever and lesions were painless and his general condition was good.

**Figure 2:**
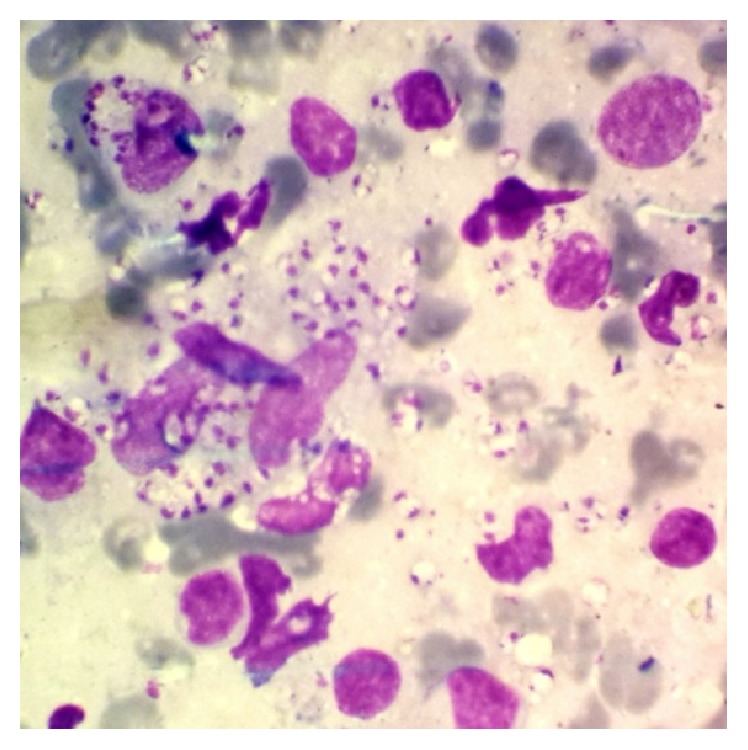
Direct smear was positive for leishman bodies (Giemsa stain).

Direct smears were prepared from the edge of skin lesions on different parts of body by using vaccinostyle and were fixed with pure methanol. Samples were stained by Giemsa 10% and examined under a light microscope and amastigote forms of* Leishmania* species were observed and CL was confirmed (Figure 2).

**Figure 3:**
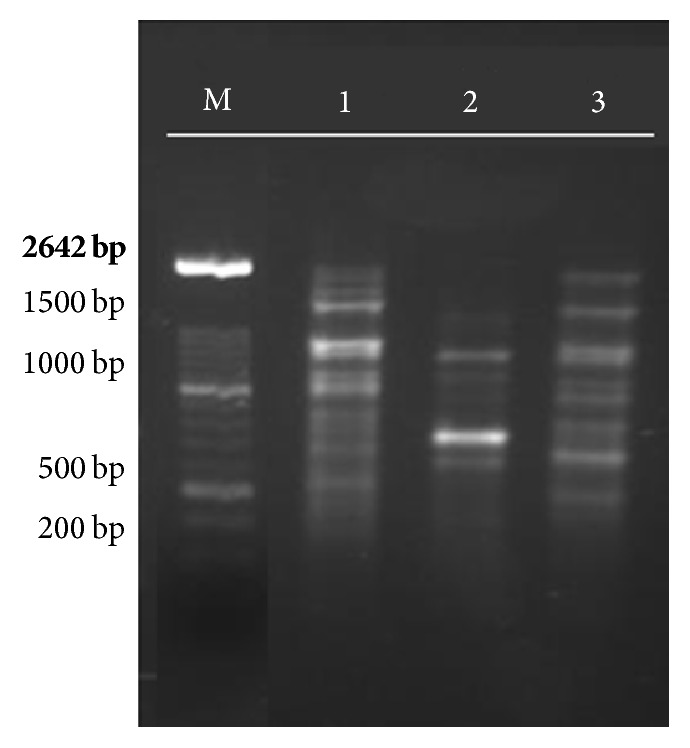
RAPD-PCR technique results by OPA4 primer in isolated strain and standards: M- marker (XIV 100 bp), (1) unknown strain, (2)* L. tropica* (MHOM/IR/IR/99), and (3)* L. major* (MHOM/IR/75/ER).

To identify the leishman agent causative of the disease materials of skin lesions cultured on RPMI-1640 medium plus 10% FBS, DNA was extracted and PCR was performed. PCR was positive for* Leishmania major* (Figure 3).

The patient was treated intramuscularly by meglumine antimoniate (Glucantime).

## 3. Discussion

This report describes an unusual case of cutaneous leishmaniasis. The patient is residing and working in a rural area in the city of Aran va Bidgol (Isfahan province, located in the center of Iran). Many farms are located near or on the wild burrows in this county. According to the development of farms and human residency in wild rodents living area in the desert region of Aran va Bidgol, CL is the most common and the disease has been endemic. In recent years Aran va Bidgol county has been reported as an important zoonotic CL focus in Isfahan province [11]. Natural leishmanial infection caused by* L. major* in great gerbil (*Rhombomys opimus*) as reservoir and* Phlebotomus papatasi* sandfly as vector was confirmed in Aran va Bidgol [11].

In the case reported in the present study, the patient had no positive history of traveling to leishmaniasis endemic areas and there were 25 lesions on his body. Of these, 8 lesions were on the face that one of them was on the upper eyelid right eye and he was suffering from ocular leishmaniasis. Untreated ocular leishmaniasis may cause ophthalmologic side effects and can cause irreparable damage. In Iran, Modarres Zadeh et al. reported four cases of ocular leishmaniasis from 1950 to 2005. In Iran two of them ended in blindness [12].* Leishmania major* with about 75% frequency is causative agent of zoonotic CL and is endemic in many rural areas of the Iran. CL caused by* L. major* is reported in 17 out of 31 provinces in Iran. Isfahan is one of the involved provinces [13, 14]. In the present study, RAPD-PCR technique showed that causative agent was* L. major*. Ocular leishmaniasis is caused by different* Leishmania* species [12]. At the present time, most cases of atypical and OL lesions of cutaneous leishmaniasis which are reported from Iran are caused by* L. major*. Hajjaran et al. reported four unusual CL cases in which lesions are more located on hands, feet, and backs [8]. Moravvej et al. described four cases of unusual cutaneous leishmaniasis in which ulcers were located all over body, face, around eyes, chin, around upper lip, chest, and back [10]. Nasiri et al. described a case of CL on lower lip [15]. Ayatollahi et al. described a case of unusual cutaneous leishmaniasis involving a solitary lesion on the right eyelid (ocular leishmaniasis) [7]. Sadeghian et al. described a case of ocular leishmaniasis on lower and upper right eyelid margins [16]. Abrishami et al. reported a case of ocular leishmaniasis on right lower lid [17]. Jafari et al. described a case of OL on upper eyelid [18]. Nikandish et al. reported OL on the right eye [19]. In other parts of the world, several cases of unusual OL cases have been reported [20–26]. OL is a rare disease and involved 2.5% of cases with CL [6, 12]. There is still no vaccine to prevent the disease and the drugs of treatment for all forms of leishmaniasis are pentavalent antimony compounds [27, 28].

Although cutaneous leishmaniasis is a self-limited disease [29], untreated OL can potentially be very serious for eyes. Since the early diagnosis and treatment of diseases can prevent the ophthalmologic side effects, ocular leishmaniasis should be considered and studied further in endemic areas of CL.
